# Post-Impact Behaviour of Pseudo-Ductile Thin-Ply Angle-Ply Hybrid Composites

**DOI:** 10.3390/ma12040579

**Published:** 2019-02-15

**Authors:** Alessia Prato, Marco L. Longana, Ambreen Hussain, Michael R. Wisnom

**Affiliations:** Bristol Composites Institute (ACCIS), University of Bristol, Queen’s Building, University Walk, Bristol BS8 1TR, UK; m.l.longana@bristol.ac.uk (M.L.L.); ah14839@my.bristol.ac.uk (A.H.); m.wisnom@bristol.ac.uk (M.R.W.)

**Keywords:** angle-ply composites, pseudo-ductility, experimental tests, Digital Image Correlation, non-destructive analysis

## Abstract

This work experimentally explores the post-impact behaviour of thin-ply angle-ply pseudo-ductile carbon fibre laminates subjected to tensile load. Indentation and low-speed impact tests were performed on standard tensile test specimens. Non-destructive tests were used to investigate the damage propagation. Digital Image Correlation (DIC) was adopted to detect the strain distribution during tensile tests. Post-damage pseudo-ductile behaviour was retained in angle-ply hybrid composites subjected to tensile loading conditions.

## 1. Introduction

Despite their excellent specific strength and stiffness, the industrial application of composite materials is still limited by their elastic-brittle behaviour. However, by acting on the composite constituents’ properties and geometry, ply thickness, architecture and lamination sequence, it is possible to achieve pseudo-ductile behaviour through a more gradual failure.

A first approach to obtain a pseudo-ductile behaviour, with a plateau on the stress-strain curve, is to use thin-ply interlaminated hybrid composites: stable and progressive fibre fragmentation in the low strain to failure material allows transfer of the load onto the higher strain to failure material without catastrophic delamination occurring [1]. Pseudo-ductile tensile response is achieved through this mechanism both in unidirectional [2] and multi-directional laminates [3]. A similar mechanism was exploited to obtain pseudo-ductile behaviour in intermingled [4], intra-ply [5], interlaminated [6] hybrid aligned discontinuous fibre composites. A second mechanism to achieve pseudo-ductility was introduced by Fuller and Wisnom [7], who modelled the use of thin-ply angle-plies to generate additional strain through a ply re-orientation mechanism, i.e., the rotation of fibres towards the loading axis combined with shear in the matrix. This mechanism has been experimentally validated [8] using plies with a cured thickness of 0.03 mm laminated in a [±Θn]_S_ lay-up (15° < Θ < 45°). It was showed through tensile test results and X-ray CT scan images, that delamination can be suppressed allowing the development of considerable pseudo-ductile strain. Yuan et al. also studied angle-ply laminates with 20-, 60- and 120-g/m^2^-thin prepreg plies and 15° and 30° angles [9] and observed the full-field fibre re-orientation with Digital Image Correlation (DIC) [10]. The two mechanisms mentioned above can be combined in a new material combination, i.e., thin-ply angle-plies, where a significant increment in pseudo-ductility can be achieved by angle re-orientation and fragmentation [8].

Thin-ply angle-plies carbon-epoxy [±26_5_]_S_, [±27_5_]_S_ and [±26_5_/0]_S_ laminates have been investigated under quasi-static cyclic loading: the first two laminates did not display any decrease in initial modulus and damage accumulation, suggesting a ductile, rather than pseudo-ductile, behaviour; the latter displayed a 20% modulus reduction, due to the gradual failure of the 0° layers [11]. Wu et al. conducted an experimental investigation on the unnotched and open-hole tensile behaviour of pseudo-ductile thin-ply angle-ply carbon fibre/epoxy [±26_5_/0]_S_ and [±25_2_/0]_S_ laminates: pseudo-ductile behaviour was observed in the unnotched specimens, and the open-hole specimens showed the same damage mechanisms, reducing the material’s notch sensitivity, as demonstrated by a combination of DIC full-field strain maps and X-ray CT scan images [12].

During their operative life, composite structures might be subjected to impact and other out-of-plane loading conditions able to generate barely visible damage that could develop into catastrophic failure [13]. In particular, low-speed impacts and indentation can produce damage on the non-impacted surface or, as internal delamination, undetectable by visual inspection. Fotouhi et al. investigated thin-ply carbon/standard-thickness glass hybrid laminates that already displayed a pseudo-ductile behaviour, subjected to indentation, demonstrating the possibility to activate new, more gradual, types of damage mechanisms [14]. Research activities on impact problems were conducted on hybrid composites by Naik et al. [15], who investigated the impact and post-impact behaviour under compressive load of a hybrid carbon/glass composite, observing a reduced notch-sensitivity in these materials in comparison to pure carbon and pure glass laminates. Kounain et al. [16] described the post-impact tensile properties of plain weave Glass Fibre Reinforced Polymer (GFRP) composite laminates and found that the residual tensile strength, strain at failure and elastic modulus decreased with the increase in the impact energy due to growth of the damage region.

This research proposes the investigation of the residual pseudo-ductility in pre-damaged angle-ply composite materials using a Tensile After Impact (TAI) technique to make a comparison with the tensile tests over undamaged specimens to estimate the presence of residual pseudo-ductility in pre-damaged specimens. The retention of pseudo-ductile behaviour after damage caused by an out-of-plane load is investigated on thin-ply angle-ply composites under tensile loading.

## 2. Materials and Specimens

The [±27_7_/0]_S_ hybrid carbon lay-up sequence proposed by Wu et al. [17] was selected as the baseline for the development of the new ([±27_7_/0]_S_)_2_ layup investigated in this research activity. This lamination sequence showed a promising combination of non-linear and pseudo-ductile behaviour as well as high strength. Moreover, the inclusion of 0° plies allows to achieve fragmentation in the composite in addition to re-orientation of the ±27° angle plies. A Skyflex UIN020 20gsm pre-preg with MR60 carbon fibres was used for the angle plies and a North M55JB 30gsm thin ply pre-preg was used for the 0° plies. Detailed information about the two pre-pregs are provided in Table 1.

The panels were cured in a vacuum bag at 120 °C. This curing cycle has been demonstrated to be suitable for both prepreg resin types, as observed in [3] and in [18]. Six sets of specimens, 280 mm long, 25 mm wide and 1.58 mm thick, were cut with a diamond saw, and 65-mm-long glass-fibre end-tabs were attached at both ends to obtain a gauge length of 150 mm.

## 3. Experimental Activity

### 3.1. Methodology Overview

Most of the characterisation work on thin-ply angle-plies laminates has been conducted in tension, and pseudo-ductility has been observed in this loading condition; the main interest in this paper is whether this behaviour is retained after impact. Therefore, although the traditional testing methodology to estimate property retention after an impact event is the Compression After Impact (CAI) test [19], this study focuses on Tension After Impact (TAI). Tensile tests (ASTM standards D3039 [20] and D5766 [21]), were performed after indentation tests (ASTM standard D6264 [22], or drop-weight, ASTM standard D7136 [23]). As the interest in pseudo-ductility has been, to date, mostly in tension, this approach was adopted to investigate property retention after a damage event, as also previously done for the effect of notches [12]. To evaluate the damage caused by the indentation and drop-weight tests, C-scans were used. The full-field strains on the specimens’ surface during the tensile tests were observed using Digital Image Correlation (DIC).

### 3.2. Indentation Tests

**Figure 1:**
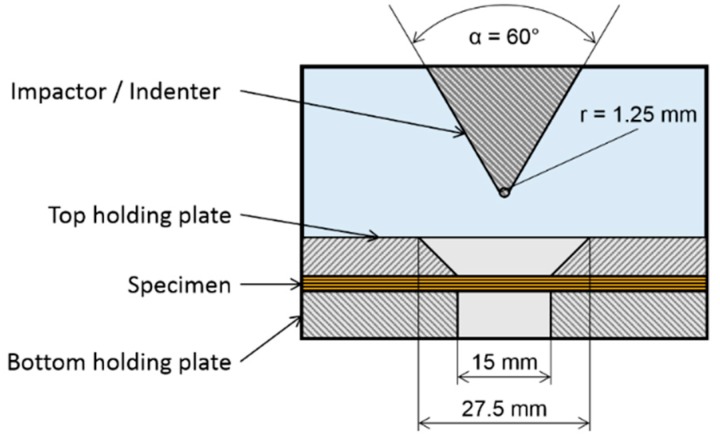
Indentation tests: specimen’s clamping system and indenter.

Indentation simulates a low-velocity impact test [22] that is run in displacement control in a quasi-static load condition. This test is used to estimate the impact energy required to obtain a similar damage state in drop-weight impact testing and for damage state observation. The test was run on a servo-hydraulic Instron 8872 test machine (Instron, Norwood, MA, USA) equipped with a 25 kN load cell at a cross-head displacement speed of 3 mm/min. A non-standard indenter with a tip radius of 1.25 mm and tip angle of 30° was used in order to produce a damage area confined to the centre of the specimen and smaller than the one produced by a standard hemispherical indenter/impactor [22,23]. An ad hoc specimen clamping system was designed to hold the specimen and allow a 15-mm-diameter damage area, as shown in Figure 1. The specimen was placed between two steel plates, machined to allow the pre-tabbed specimens to be supported throughout, and bolted with a torque wrench to ensure that the same boundary conditions were repeated each time.

**Figure 2:**
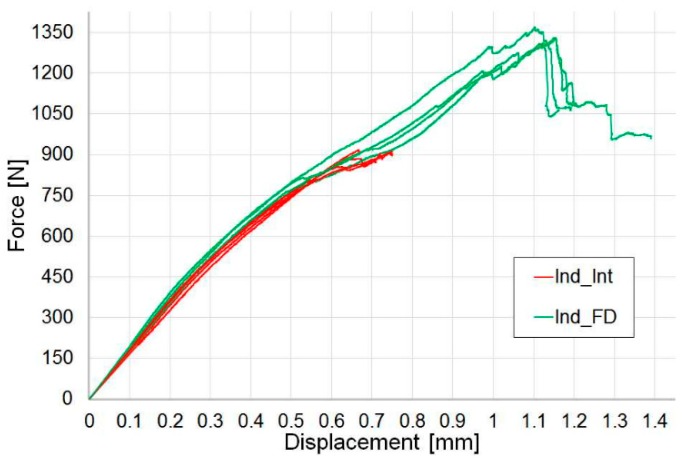
Indentation tests: load-displacement curves of Indented Fully Damaged (Ind_FD) and Indented Interrupted (Ind_Int) specimens.

Two sets of specimens were tested with this set-up. One set was tested until complete impactor penetration was achieved, identifiable on the force-displacement curve as a sudden load drop, see Indented Fully Damaged (Ind_FD) curves in Figure 2. The second set of tests was interrupted at ⅔ of the average peak load needed to fully damage the specimens, see Indented Interrupted (Ind_Int) test curves in Figure 2.

The load-displacement curves are very consistent. In the Ind_FD specimens’ curves, three regions can be identified:

- an elastic deformation region, between 0 and 750 N
- a damage initiation and evolution region, identifiable with a slope change in the force-displacement curve between 750 and the sudden load drop around 1300 N
- final indenter penetration region

As shown in Figure 2, Ind_Int tests were deliberately stopped after damage onset.

### 3.3. Impact Tests

**Figure 3:**
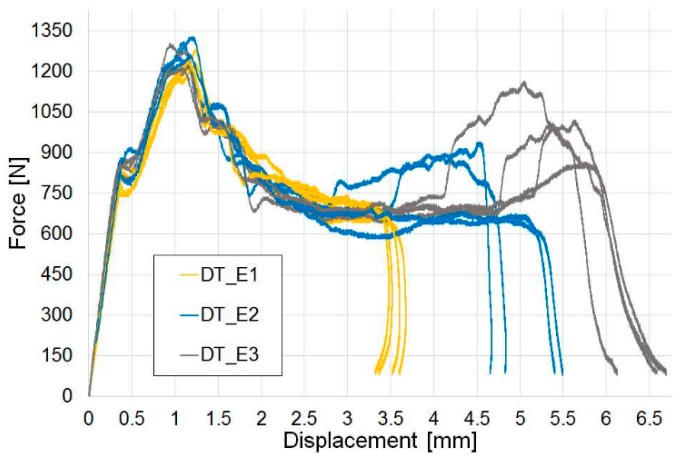
Drop tower impact tests: load-displacement curves for different energy levels: 2.2 J (DT_E1), 3.2 J (DT_E2) and 4.2 J (DT_E3), respectively.

The impact tests [23] were run with an Instron Dynatup 9250HV impact tower (Instron, Norwood, MA, USA). A 15-kN piezo-electric load cell was used to acquire the load data. A light-gate sensor was mounted on the drop tower to estimate the drop-weight velocity as a flag mounted on the falling-weight passes through it. The displacement is indirectly measured by the drop tower as numerical integration of the velocity [24]. The impactor and the specimen clamping system are the same as the one used for the indentation tests. Based on the indentation tests results, three different nominal impact energies have been used, i.e., 2.2 J, 3.2 J and 4.2 J, to impact three sets of specimens, i.e., DT_E1, DT_E2 and DT_E3, respectively. Lower values of energy could not be reliably obtained due to equipment limitations. These impact energies allow to generate a damage region in agreement with that obtained with the indentation tests. The load-displacement curves for the impact tests are shown in Figure 3.

### 3.4. Post-Indentation and Post-Impact Damage Analysis

**Figure 4:**
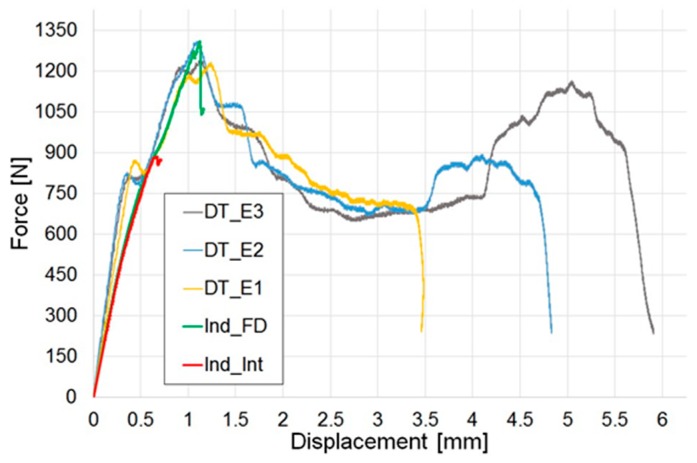
Indentation and impact tests: comparison of the force–displacement curves of representative tests. Indented Fully Damaged (Ind_FD) and Indented Interrupted (Ind_Int) specimens and samples impacted in the drop tower at 2.2 J (DT_E1), 3.2 J (DT_E2) and 4.2 J (DT_E3).

**Figure 5:**
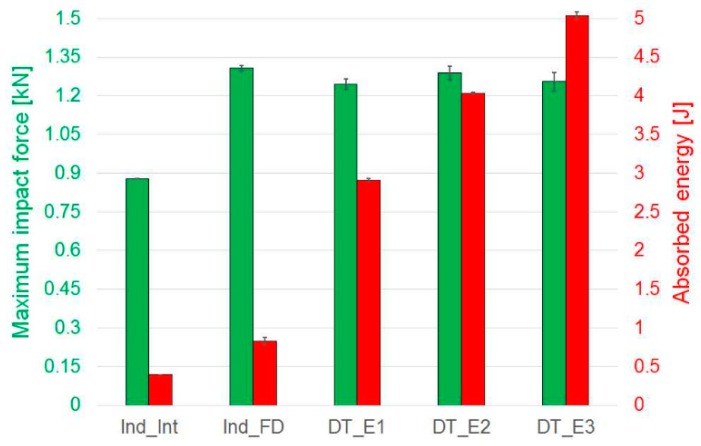
Indentation and impact tests: comparison of peak of force and absorbed energy. Indented Interrupted (Ind_Int) and Indented Fully Damaged (Ind_FD) specimens and samples impacted respectively at 2.2 J (DT_E1), 3.2 J (DT_E2) and 4.2 J (DT_E3). Error bars represent standard deviation.

A comparison of the representative force-displacement curves of indentation and impact tests is presented in Figure 4. Within the tests performed in the same static or dynamic test configuration, there is a correlation in the linear elastic region of the curves. The difference of slope in the elastic deformation between indented and impacted tests could be explained considering that in the indentation tests, the displacement is directly measured through the cross-head displacement, whilst in the impact tests, it is indirectly calculated as described above. Moreover, the strain-rate effect, i.e., dynamic loading condition for the drop tower tests and quasi-static for the indentation, might contribute to this difference. The peak force of the representative Indented Fully Damaged graph (Ind_FD) is similar to the maximum value obtained for the drop tower tests. A comparison of the peak of force is also shown in Figure 5.

The amount of energy absorbed by the material during indentation and impact during the development of specific failure modes and subsequent damage propagation is another parameter of interest, especially for impact and crash problems. The energy absorbed, i.e., the area underneath the force–displacement curves, during all the tests is summarised below in Figure 5. A good repeatability of test results is shown. As expected, as the impact energy increases, the absorbed energy also increases.

An additional comparison refers to the damaged area. The analysis was performed using an ultrasound pulse-echo C-scan, single transmitter–receiver focused probe. The images obtained as output, provide an approximate representation of the damage extent. Table 2 summarises the C-scan set-up parameters.

**Figure 6:**
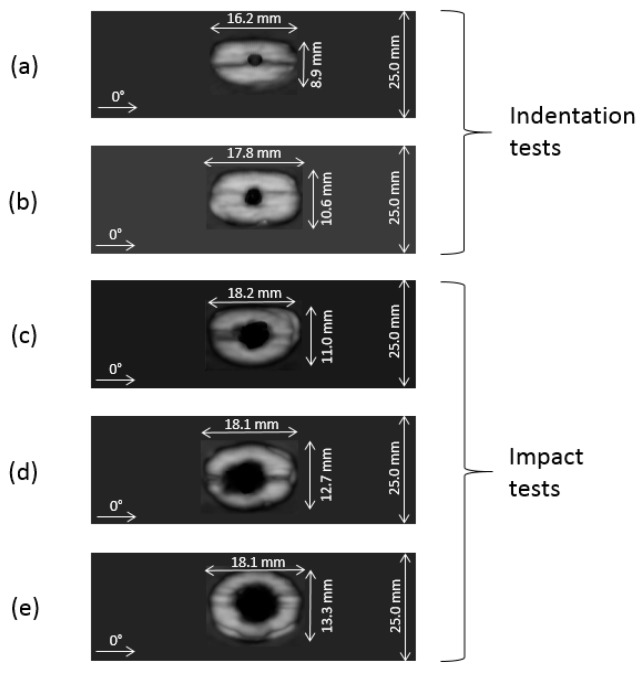
C-scan: images of reference damaged specimens. (**a**) Indented Interrupted (Ind_Int), (**b**) Indented Fully Damaged (Ind_FD), (**c**) impact at 2.2 J (DT_E1), (**d**) impact at 3.2 J (DT_E2), (**e**) impact at 4.2 J (DT_E3).

**Figure 7:**
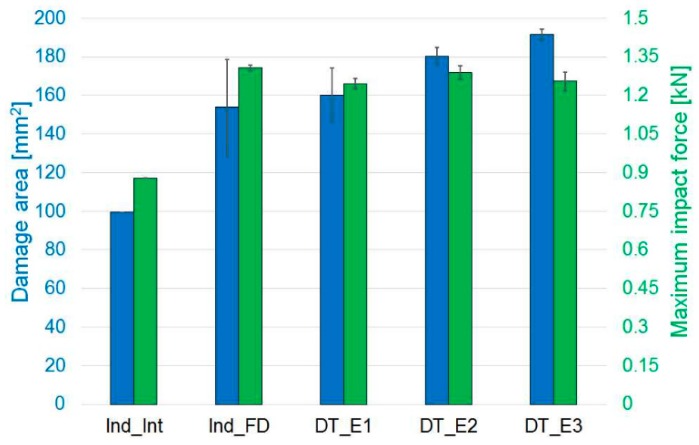
Indentation and impact tests: comparison of damage area and maximum impact force. Indented Interrupted (Ind_Int) and Indented Fully Damaged (Ind_FD) specimens and samples impacted respectively at 2.2 J (DT_E1), 3.2 J (DT_E2) and 4.2 J (DT_E3). Error bars represent standard deviation.

From the results, see Figure 6 and Figure 7, for the pre-indented specimens, the damage increases as the indenter penetrates the material. The force-displacement Indented Interrupted (Ind_Int) test curves show a lower peak force that is related to a reduced damage area in these specimens with respect to the Indented Fully Damaged specimens (Ind_FD), tested until complete penetration of the indenter was achieved.

**Figure 8:**
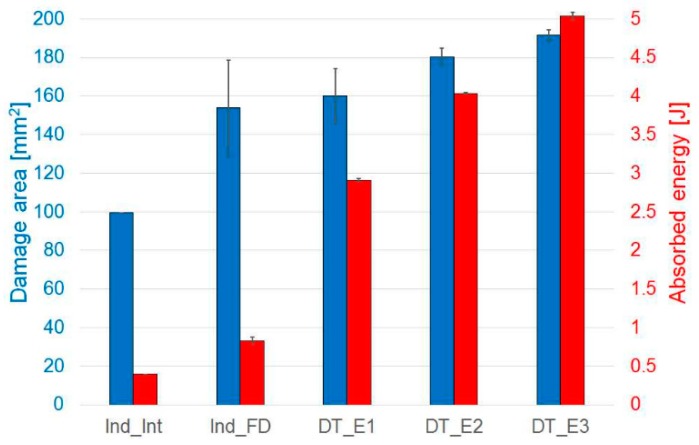
Indentation and impact tests: comparison of damage area and absorbed energy. Indented Interrupted (Ind_Int) and Indented Fully Damaged (Ind_FD) specimens and samples impacted respectively at 2.2 J (DT_E1), 3.2 J (DT_E2) and 4.2 J (DT_E3). Error bars represent standard deviation.

The pre-impacted specimens, instead, showed a damaged region of almost the same dimension in all the tests, even though progressively higher energies were used, Figure 8. The use of an ad hoc designed clamping system, with 15-mm-diameter windows for impact and indentation, allows to limit the damage in the material and, at the same time, created a constraint on the delamination area propagation that, however, is in all cases close to half the specimen width. Figure 7 shows the relation between damage area and maximum impact force: the damage area increased as the impact force increased, remaining anyway limited because of the mechanisms mentioned above. Figure 8 presents the relationship between damage area and absorbed energy: as the impact energy increased, the absorbed energy increased accordingly as well as the delamination region around the indented/impact point, which remained limited due to the geometrical constraint imposed by the clamping system, as previously mentioned.

In all the cases, the longer dimension of the damage is in the longitudinal direction of the specimen and the limited transversal propagation can be related also to the re-orientation of the angle-plies towards the 0° direction or, more probably, to the use of material with a lower modulus.

### 3.5. Tensile Tests

Tensile tests have been used to evaluate the residual pseudo-ductile behaviour of these laminates. Five sets of specimens, damaged by indentation and impact, were tested along with an undamaged set as a benchmark. A servo-hydraulic Instron 8772 universal test machine (Instron, Norwood, MA, USA), equipped with a 100-kN load cell, was used to load the specimens at a cross-head displacement speed of 1 mm/min. A LaVision Stereo DIC system was used to obtain full-field strain maps over the specimen surface and assess the damage development around the indented/impacted area during the tensile test loading. Table 3 summarises the Stereo DIC set-up and the processing parameters.

#### 3.5.1. Benchmark Tensile Test Results

**Figure 9:**
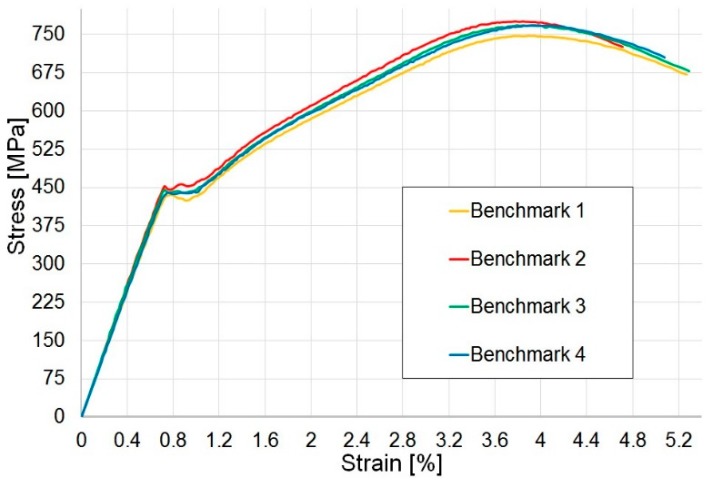
Tensile tests: undamaged benchmark results.

The stress–strain results of the benchmark specimens in Figure 9 show clearly four different regions:

- an initial linear elastic region up to the pseudo-yield point
- a short plateau, with constant stress where fragmentation occurs
- a pseudo-plastic region, with continuous increment of the stress, where angle re-orientation in the material occurs, up to the peak of stress
- stress reduction towards the complete failure of the laminate

In these tests, a pseudo-ductile response is clearly visible, with an average pseudo-ductile strain, calculated with the definition proposed by Wisnom et al. [7], i.e., the difference between the failure strain and the elastic strain calculated from the initial modulus at the same stress, equal to 3.9% with low dispersion of data.

#### 3.5.2. Post-Indentation Tensile Test Results

**Figure 10:**
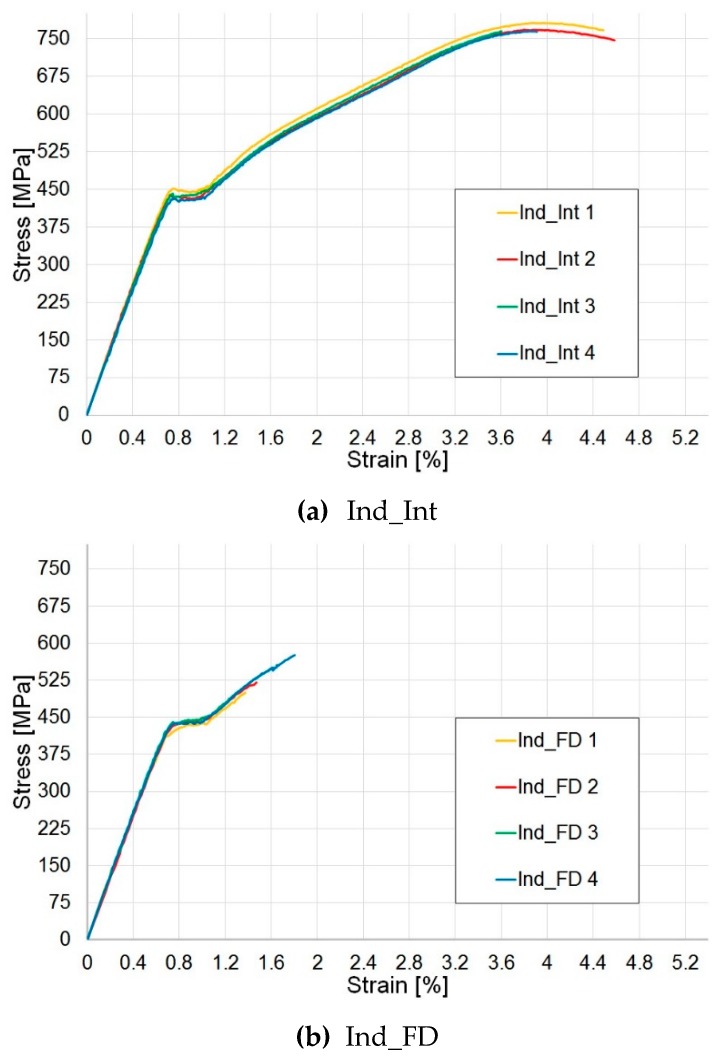
Tensile tests: post-indentation results. (**a**) Indented Interrupted (Ind_Int) and (**b**) Indented Fully Damaged (Ind_FD) specimens.

The post-indentation tensile test results (Figure 10) present a global pseudo-ductile response. In the stress–strain curves, the maximum stress is higher in the Indented Interrupted (Ind_Int) specimens, compared with the Indented Fully Damaged (Ind_FD) samples due to the presence of a smaller damage region (see Section 3.4). The knee point occurred at the same stress value as for the benchmark specimens.

#### 3.5.3. Post-Impact Tensile Test Results

**Figure 11:**
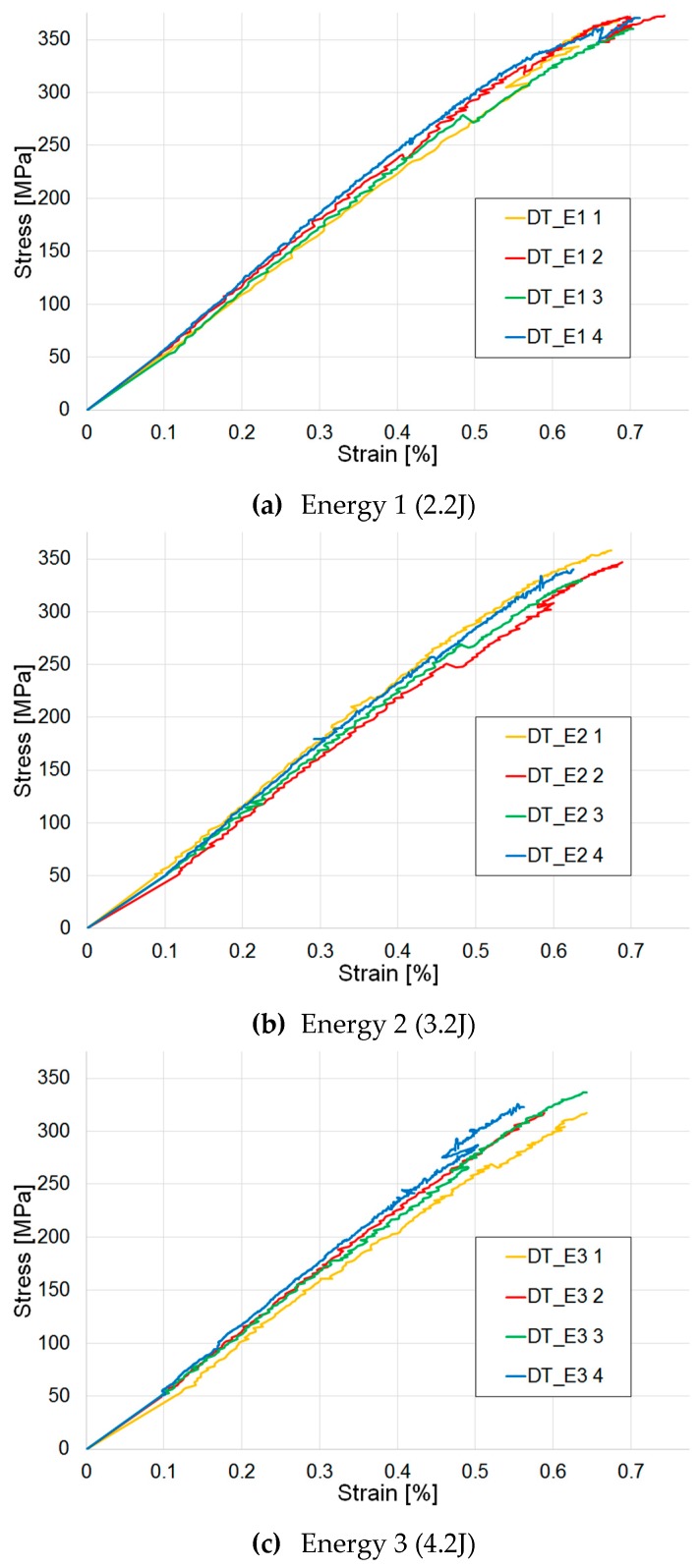
Tensile tests: post-impact results. (**a**) samples impacted at 2.2 J (DT_E1), (**b**) samples impacted at 3.2 J (DT_E2), (**c**) samples impacted at 4.2 J (DT_E3).

No global pseudo-ductile behaviour can be observed in the post-impact tensile test results (Figure 11). An almost linear elastic response is shown, with no pseudo-yield point or stress plateau or subsequent pseudo-plastic behaviour. No great differences can be noticed between the curves, and the initial elastic response is the same for all three sets of data. The maximum stress value slightly decreases as the impact energy level increases due to the high value of initial impact energy from the drop tower, which is higher than the energy absorbed during the indentation tests. The lack of a global pseudo ductile response can be explained considering that the high value of energy imposed by the drop tower, which caused the development of extensive damage, delamination and consequently the impossibility for the pseudo-ductile damage mechanisms to take place.

## 4. Discussion

Some additional considerations are required to better understand the results of all the tests.

**Figure 12:**
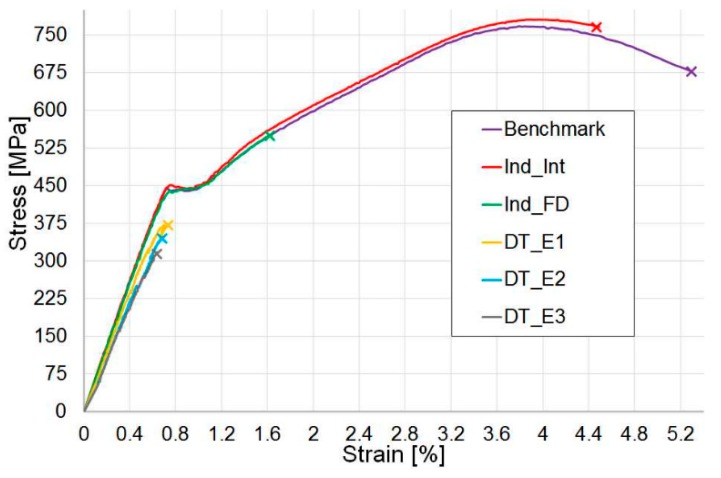
Tensile stress–strain curves: a comparison. Benchmark (undamaged specimens), pre-Indented Fully Damaged (Ind_FD) and pre-Indented Interrupted (Ind_Int) specimens and samples pre-impacted at 2.2 J (DT_E1), 3.2 J (DT_E2) and 4.2 J (DT_E3).

Figure 12 proposes a comparison of the stress–strain curves for typical specimens in all the test configurations investigated: Benchmark (undamaged specimens), Indented Fully Damaged (Ind_FD) and Indented Interrupted (Ind_Int) specimens and samples impacted at 2.2 J (DT_E1), 3.2 J (DT_E2) and 4.2 J (DT_E3). The mechanical response of the benchmark and the indented interrupted tests is almost the same, as only a small amount of damage was produced in the indented specimens. On the contrary, for the indented fully damaged and all the drop-tower impact tests, where a more extended initial damage is present, the response, before final catastrophic failure, is solely linear-elastic.

**Figure 13:**
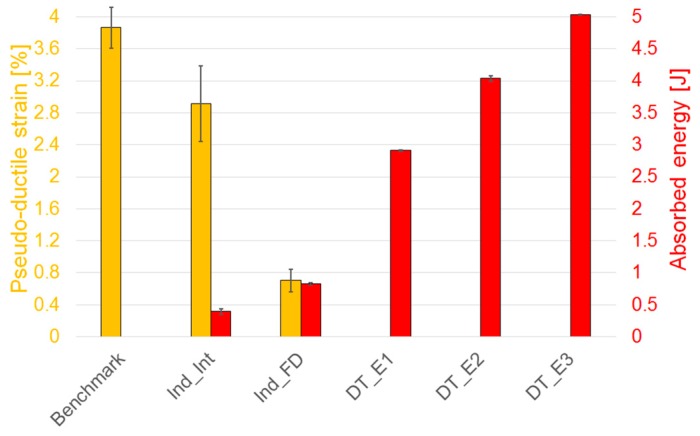
Pseudo-ductile strain and absorbed energy: a comparison. Benchmark (undamaged specimens), Indented Interrupted (Ind_Int), Indented Fully Damaged (Ind_FD) specimens and samples impacted respectively at 2.2 J (DT_E1), 3.2 J (DT_E2) and 4.2 J (DT_E3). Error bars represent standard deviation.

Figure 13 summarises the absorbed energy during indentation and impact and the pseudo-ductile strain values computed from the tensile tests in all the different sets of data considered in this research. The absorbed energy increases as the impact or indentation energy level increases, with a maximum for the DT_E3 specimens, impacted at the highest energy equal to 5 J and lower values in the other tests. The pseudo-ductile strain is maximum in the benchmark specimens, decreasing slightly for the Interrupted Indented (Ind_Int) specimens and substantially absent for the Indented Fully Damaged (Ind_FD) specimens. No global pseudo-ductile response is apparent in the post-impacted specimens due to the presence of a reduced effective net section as a result of the damage during the impact test. It is related to the much larger damage caused by the penetration of the specimens by the impactor for the tests performed at higher impact energy. This caused delamination around the impact point and failure in the adjacent high-strain fibres that led to an earlier catastrophic failure of the specimens under tensile loads.

However, although a global pseudo-ductile response is observed only on the indented specimens, a careful analysis of the full field strain obtained with the Digital Image Correlation (DIC) demonstrates the presence of localised areas of pseudo-ductile behaviour around the damaged region.

**Figure 14:**
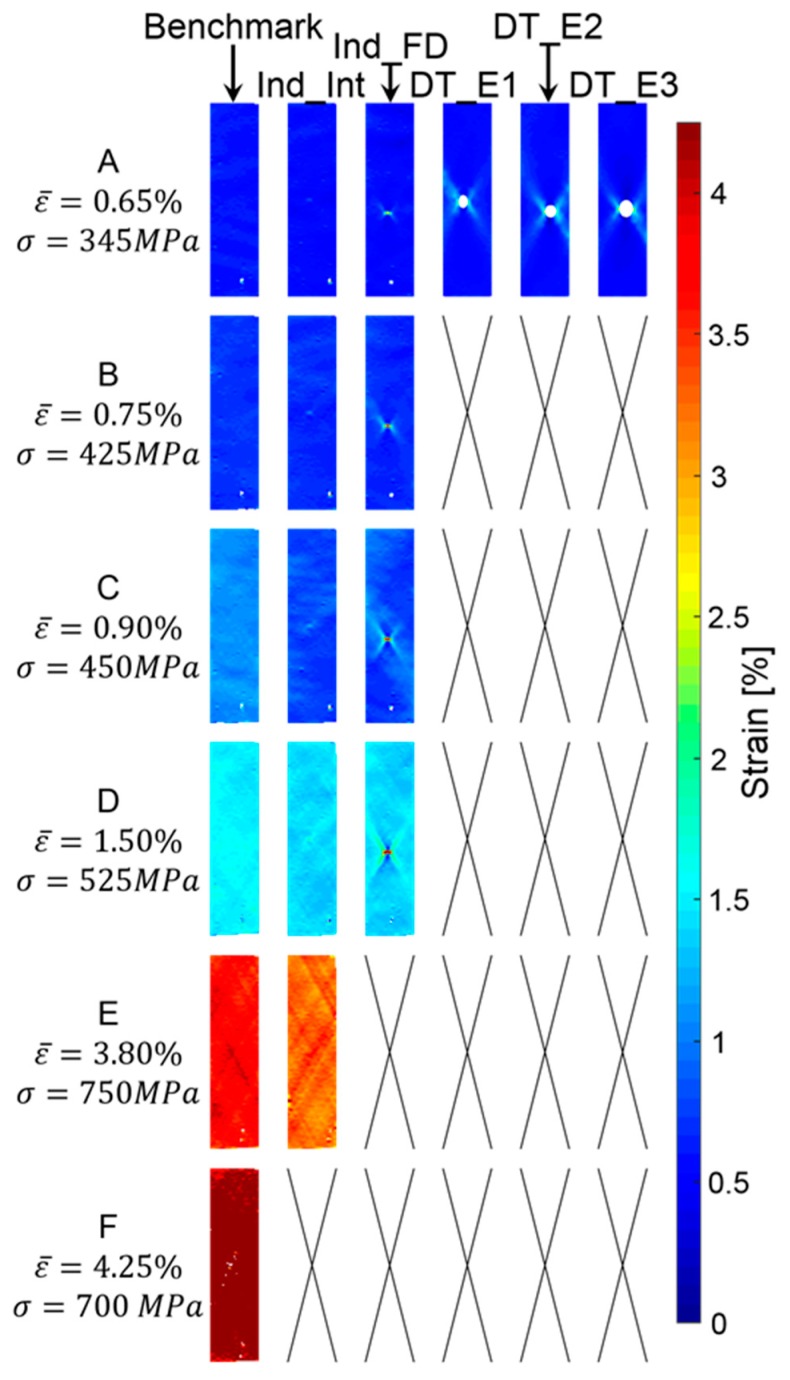
Full tensile strain field: a comparison. Benchmark (undamaged specimens), Indented Fully Damaged (Ind_FD) and Indented Interrupted (Ind_Int) specimens and samples impacted at 2.2 J (DT_E1), 3.2 J (DT_E2) and 4.2 J (DT_E3).

**Figure 15:**
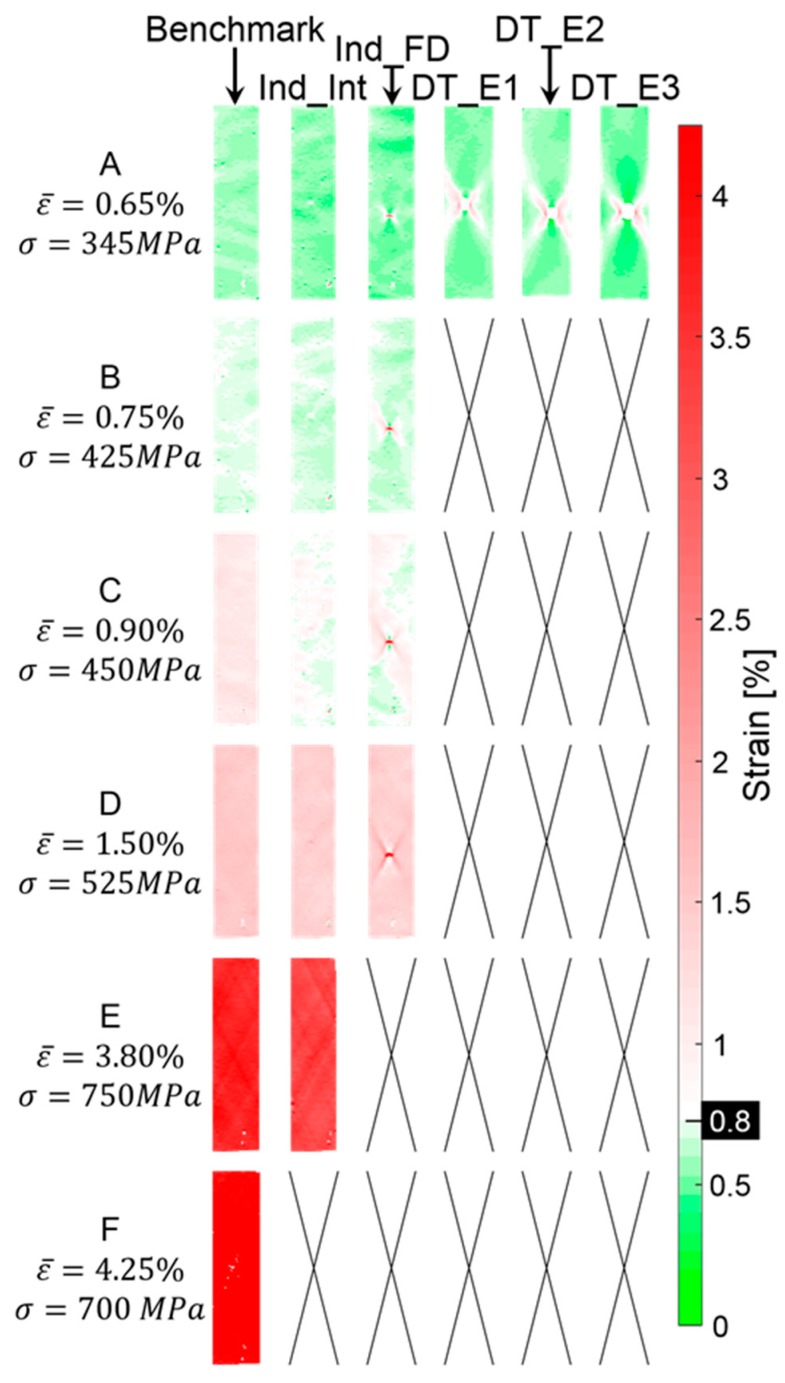
Tensile strain field referred to threshold: a comparison. Benchmark (undamaged specimens), Indented Fully Damaged (Ind_FD) and Indented Interrupted (Ind_Int) specimens and samples impacted at 2.2 J (DT_E1), 3.2 J (DT_E2) and 4.2 J (DT_E3).

Figure 14 and Figure 15 show the longitudinal surface strain maps obtained with the DIC for representative specimens in all the tested sets: the benchmark, pre-indented and pre-impacted samples measured at different stress-strain levels. In Figure 14, a traditional 256-colour map has been used to represent the full field strain distribution. In Figure 15, a two-colour map has been used, and the threshold value between the two regions is represented by the failure strain of the M55 material used in the 0° layers, i.e., 0.8%.

In Figure 15, it can be observed that at an applied global strain equal to 0.65% of all the post-impacted specimens show a region, close to the impacted area, where the strain exceeds the threshold value. A similar result is shown in the pre-indented Ind_FD samples. Different results instead occurred for the pre-indented Ind_Int tests and the benchmark set, where, in the DIC image, the strain does not exceed the threshold anywhere.

As the strain increases, no additional results are available for the pre-impacted specimens due to complete failure, whilst the benchmark and all the pre-indented sets show that pseudo-ductility must have occurred locally after a strain level equal to 0.75%. A more uniform distribution of the strain is visible in the Ind_Int tests due to a reduced stress concentration.

**Figure 16:**
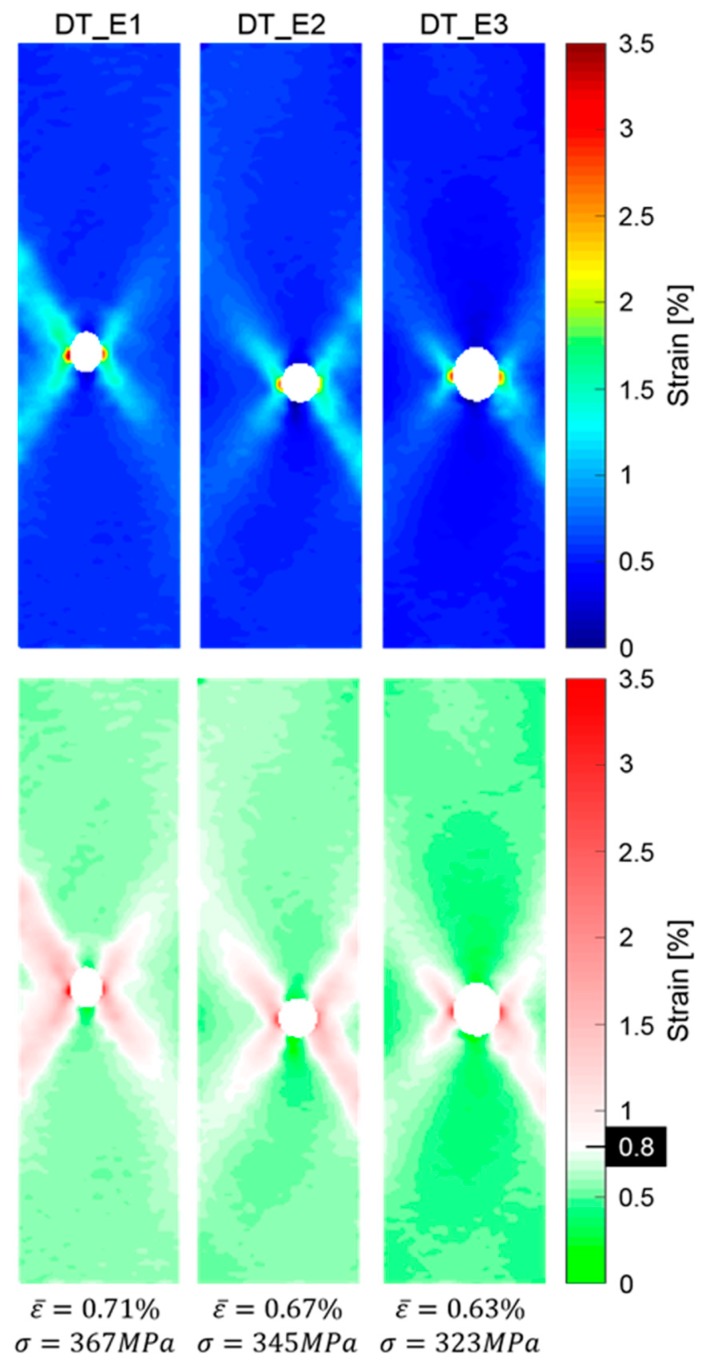
Post-impact tensile tests: full tensile strain field and threshold strain. Samples impacted at 2.2 J (DT_E1), 3.2 J (DT_E2) and 4.2 J (DT_E3).

Figure 16 shows the post-impact results for the three different impact energy levels and the DIC longitudinal strain maps just before complete failure of the reference specimens. The full-field strain distribution, Figure 16 (top), presents average strain values lower than 1%. Referring to the same images, but with the M55 strain failure value of 0.8% used as a threshold, Figure 16 (bottom), it can be observed that in all cases, a region with a strain value higher than the benchmark pseudo-yield strain is visible in all the sets. The geometry of this region tends to be aligned with the ±27°direction of the angle-ply carbon fibres, as in the failure mode. From that, it is possible to say that pseudo-ductility occurs during tensile tests also in all the pre-impacted set of specimens in a localised area around the impacted region.

In all the cases, a post-indentation and post-impact pseudo-ductile response has been shown under tensile loading in the proposed thin-ply angle-ply hybrid carbon fibre composites. The behaviour obtained is in accordance with that shown by Wu et al. [12], who demonstrated that pseudo-ductility was achieved in IM-HM [±25_2_/0]_S_ laminates and that notch-sensitivity can be reduced in these thin-ply laminates by taking advantage of the fragmentation, delamination and angle-ply re-orientation damage mechanisms.

## 5. Conclusions

In this research activity, the post-impact behaviour of a [±27_7_/0]_S_ thin-ply angle-ply hybrid carbon fibre laminates was studied to evaluate the residual pseudo-ductile behaviour in tension.

Five different tests, i.e., Indented Fully Damaged (Ind_FD), Indented Interrupted (Ind_Int), impact at 2.2 J (DT_E1), at 3.2 J (DT_E2) and at 4.2 J (DT_E3), were used to pre-damage the specimens. C-scanning was used to evaluate the delamination region inside the specimens caused by the different pre-damage loads. Tensile tests were performed on an undamaged benchmark and pre-damaged specimens to investigate the residual global and local pseudo-ductile response of the material. DIC was used during tensile tests to estimate the strain fields.

It was concluded that:

- in both indentation and impact tests, a good repeatability of the force-displacement curves was shown. Moreover, while the different pre-damaging tests caused changes in the absorbed energy, the maximum applied force remained almost the same for the fully indented and all the impacted specimens.
- the C-scan analysis showed the damage area, specifically delamination, obtained as a consequence of indentation and impact. The damage area progressively increased as the impact and absorbed energy increased; however, it was restrained by the ad hoc designed clamping system.
- a 3.9% pseudo-ductile strain was obtained for the benchmark [±27_7_/0]_S_ thin-ply angle-ply hybrid carbon fibre laminates.
- in both post-indentation and post-impact tensile tests, the investigated lay-up displayed a residual pseudo-ductile behaviour:
  ○ the indented specimens displayed a global pseudo-ductile behaviour. Pseudo-ductile strains of 2.9% and 0.7 % were achieved in the Indented Interrupted (Ind_Int) and Indented Fully Damaged (Ind_FD) specimens, respectively.
  ○ for the post-impact specimens, no global pseudo-ductile behaviour was observed. However, the DIC analysis showed that, locally, the strain exceeded the pseudo-yield strain calculated from the benchmark test, meaning that a pseudo-ductile behaviour was achieved also for this pre-damage configuration near the hole.

To conclude, it has been demonstrated that pre-damaged thin-ply angle-ply hybrids retain at least local tensile pseudo-ductility. As the proposed un-damaged layup showed a beneficial progressive failure and an additional pseudo-ductile stress–strain response before catastrophic failure, the obtained results are particularly relevant for the design of composites subjected to impact loading conditions.

## Figures and Tables

**Table 1:** Fibres and cured plies main properties.

	Skyflex UIN020 [3]	North M55JB/epoxy [18]
**Fibres properties**		
Carbon fibres type	Pyrofil MR60H 24P (12k)	Toray M55JB
Manufacturer	Mitsubishi Rayon	Toray
Elastic modulus (GPa)	290	540
Strain to failure (%)	1.9	0.8
Tensile strength (MPa)	5.68	4.02
Density (kg/m^3^)	1.81	1.91
**Cured ply properties**		
Manufacturer	Skyflex-SK Chemicals	North TPT
Fibre volume fraction (%)	47.8	51.3
Cured ply thickness (µm)	25.4	30.8
Initial modulus (GPa)	140.4	277.1

**Table 2:** C-scan main parameters.

Characteristics	Set-Up
Probe designation	Olympus 3.0’’ Spherical PTF
Frequency (MHz)	10
Focal length (mm)	76.2

**Table 3:** Stereo DIC set-up and the processing parameters.

Characteristic	Set-Up
Technique used	Stereo DIC
Software	8.4.0
Subset size (pixels)	51
Step size (pixels)	20
Camera	Imager LX 16MP
Lens	Tokina ATX AF 100/2.8
Frame rate	2
Image Resolution (pixels)	3603 (w) × 5012 (h)
Field of view (mm)	71.76 (w) × 99.83 (h)
Spatial resolution (mm)	0.398
Displacement resolution (mm)	0.53
Strain resolution (µε)	79
